# Distribution of Urea in the Body

**Published:** 1915-06

**Authors:** 


**Distribution of Urea in the Body.** D. M. Davis, Johns Hopkins Hosp. Bull., May 1915, considers the urease method described by E. K. Marshall, Jr., as just as accurate for the body fluids and tissues as for urine and as the only method available practically for comparative determinations. In supposedly normal dogs, he found the urea content of blood, blood serum, bile and various viscera and muscles as approximately equal, being about 25 m.g. per 100 G. or C.C. in one dog, about 20 in the other. Fatty tissues as omentum, of course show much lower quantities, approximately 5 m.g. On the other hand, the urinary organs show far higher figures, e.g., 52 for prostate and urethra, 164 for bladder, 159-221 for kidney. On injecting 20 G. of urea, the figures rose from 21-24 for blood serum, liver and muscles to 493 for blood serum, and 354-409 for organs. In another dog, injected and ureters ligated, the figures averaged about 800.

